# Systemic metabolic, hormonal, and glycomic remodeling during a 72-hour fast in healthy adults: a pilot study

**DOI:** 10.3325/cmj.2026.67.226

**Published:** 2026-06

**Authors:** Gordan Lauc, Petar Brlek, Luka Bulić, Nina Šimunić Briški, Jelena Šimunović, Ivana Duvnjak Orešković, Lara Butumović, Damir Marjanović, Danijela Klarić, Ana Petrović, Jan Tesla Frcko, Dragan Primorac

**Affiliations:** 1 Genos Glycoscience Research Laboratory, Zagreb, Croatia; 2 Faculty of Pharmacy and Biochemistry, University of Zagreb, Zagreb, Croatia; 3 St. Catherine Specialty Hospital, Zagreb, Croatia; 4 School of Medicine, Josip Juraj Strossmayer University of Osijek, Osijek, Croatia; 5 Department of Molecular Biology, Faculty of Science, University of Zagreb, Zagreb, Croatia; 6 International Burch University, Sarajevo, Bosnia and Herzegovina; 7 Institute for Anthropological Research, Zagreb, Croatia; 8 Faculty of Dental Medicine and Health, Josip Juraj Strossmayer University of Osijek, Osijek, Croatia; 9 Department of Biochemistry & Molecular Biology, Forensic Science Program, The Pennsylvania State University, State College, PA; 10 The Henry C. Lee College of Criminal Justice and Forensic Sciences, University of New Haven, West Haven, CT; 11 Sana Kliniken Oberfranken, Coburg, Germany; 12 School of Medicine, University of Split, Split, Croatia; 13 Medical School, University of Rijeka, Rijeka, Croatia; 14 Medical School, University of Mostar, Mostar, Bosnia and Herzegovina; 15 National Forensic Sciences University, Gandhinagar, India; 16 University of Pittsburgh, School of Medicine, Pittsburgh, PA

## Abstract

**Aim:**

To investigate integrated biochemical and glycomic signatures in humans related to coordinated metabolic and endocrine adaptations during prolonged fasting, which are essential for maintaining systemic homeostasis.

**Methods:**

This single-arm longitudinal interventional study enrolled five healthy adults who underwent a 72-hour water-only fast. Blood samples were collected at baseline (T0), immediately after fasting (T1), and after 11 days of refeeding (T2). A broad panel of biochemical, hormonal, inflammatory, and glycomic parameters was determined. Time-dependent differences were evaluated using the Friedman test.

**Results:**

Fasting induced a distinct biphasic response across multiple circulating markers, with significant alterations in total cholesterol, C-reactive protein, thyroid-stimulating hormone, and free triiodothyronine at T1 followed by recovery toward baseline at T2. Insulin and glucose concentrations declined during fasting and increased after refeeding, although these changes did not reach statistical significance. Plasma, immunoglobulin G (IgG), and IgA N-glycosylation profiles were extensively remodeled, which indicated dynamic metabolic and immune system adaptation. Liver enzymes, electrolytes, and most lipid fractions exhibited only minor and reversible fluctuations.

**Conclusion:**

A 72-hour fast was associated with metabolic and hormonal changes consistent with an environment conducive to autophagy, although autophagy was not measured directly. The protocol appeared feasible and well tolerated in a small cohort of healthy adults. Given the limited sample size, these findings should be considered preliminary and require confirmation in larger studies incorporating direct autophagy markers and additional time points.

Nutrient deprivation has long been a part of human life – occurring during periods of food scarcity, practiced for cultural or religious reasons, or used for medical purposes. Medical applications of fasting arise from its positive effects on weight loss and metabolic biomarkers, such as glucose and insulin (1). As obesity is currently considered a global pandemic and a major risk factor for numerous metabolic and cardiovascular disorders, the importance of high-quality treatments for these conditions is growing rapidly (2,3). Beyond these well-known systemic effects, fasting also stimulates autophagy, a process that has recently gained considerable scientific attention. In order to improve current therapeutic approaches, we need to completely understand metabolic changes occurring during fasting.

Like any other condition that disrupts homeostasis, nutrient deprivation leads to the activation and deactivation of numerous biological mechanisms, with the purpose of maintaining dynamic equilibrium in the body. After 12-24 hours of fasting, glycogen storage depletes and gluconeogenesis remains the main source of glucose (4). However, this is compensated for by a metabolic switch to lipid energy sources through lipolysis. Free fatty acids (FFAs) directly serve as cellular fuel in certain tissues through mitochondrial β-oxidation, as well as a source of acetyl-CoA, which is used for the production of ketone bodies in ketogenesis (5). Glucose is preserved for the brain and other glucose-dependent tissues through decreased insulin and increased counterregulatory hormones (glucagon, catecholamines, and cortisol), which reduces insulin sensitivity and inhibits peripheral glucose uptake (6).

However, at the cellular level, these processes become far more complex. Fasting-induced metabolic changes are controlled by sophisticated regulatory mechanisms, including autophagy. Autophagy is a catabolic process that occurs under normal physiological conditions and under various forms of cellular stress (hypoxia, infection, or nutrient deprivation) (7). It involves the formation of double-membrane vesicles, referred to as autophagosomes, which sequester cellular content and fuse with lysosomes to form autolysosomes. Within these structures, lysosomal enzymes degrade misfolded or aggregated proteins and damaged cell organelles into basic molecular components (8,9). This is crucial for preserving the balance between macromolecule synthesis and degradation, especially under stress, when cells are deprived of essential precursors for macromolecule synthesis (10). In addition to its role in cellular recycling, autophagy also interacts with cell death pathways, either supporting cell survival under stress or, in certain contexts, contributing to autophagy-associated regulated cell death (8). Importantly, dysregulated autophagy has been associated with neurodegenerative diseases, inflammatory diseases, metabolic disorders and cancer, which highlights its potential therapeutic relevance (11-13).

Previous studies have examined metabolic changes during fasting, often focusing on specific fields of medicine. Browning et al analyzed changes in plasma metabolite concentrations during a 48-hour fast, primarily focusing on sexual dimorphism (14). Jiang et al investigated metabolic changes during a five-day water-only fast in 45 healthy individuals, assessing metabolic-syndrome and thyroid-related biomarkers and reevaluating them after refeeding (15). However, no studies have evaluated plasma metabolite changes in the context of autophagy. Current research on autophagy and fasting mainly revolves around autophagic flux – an increase in autophagosomes, gene expression, and the activity of signaling pathways relevant to autophagy under nutrient restriction (16-19). Importantly, Fritzen et al examined the effects of exercise and insulin stimulation on autophagy in human muscle, confirming that insulin was a potent inhibitor of autophagy in this tissue (20).

Therefore, the aim of this study was to assess metabolic changes during prolonged fasting in order to elucidate the metabolic, hormonal, and glycan dynamics of fasting. To achieve this, we assessed changes in selected biochemical and glycan parameters after 72 hours of fasting, which allowed us to examine metabolic changes occurring as a fasting response.

## Participants and methods

This was a single-arm interventional pilot study with a longitudinal design. In December 2025, the study enrolled five volunteer participants (four male and one female). The participants were White and aged from 40 to 65 years. Individuals with acute or chronic diseases or undergoing long-term or ongoing treatment were not included. Participants underwent a 72-hour water-only fast. Blood samples were taken at three time points: a baseline measurement before fasting (T0), a measurement after the fast was complete (T1), and a control measurement 11 days after the end of the fast (T2). The samples were taken in the morning. Biochemical parameters included liver and muscle enzymes (alanine transaminase [ALT], aspartate transaminase [AST], gamma-glutamyl transferase [GGT], alkaline phosphatase [ALP], lactate dehydrogenase [LDH], and creatine kinase [CK]), glucose metabolism (glucose and insulin), lipid profile (total cholesterol, triglycerides, HDL-cholesterol, LDL-cholesterol), electrolytes (potassium and sodium), inflammatory markers (C-reactive protein), and hormones (thyroid-stimulating hormone [TSH], free triiodothyronine [fT3], free thyroxine [fT4], and cortisol). Additionally, IgG, IgA, and plasma glycome profiles were determined. The Friedman test was used to assess the differences across the time points, with the significance level set at 0.05. Statistical analysis was conducted using the JASP statistical software. The study was conducted in accordance with the Declaration of Helsinki and approved by the Ethics Committee of St. Catherine Specialty Hospital (25/29-I).

### IgG N-glycome analysis

Plasma samples obtained were randomly assigned across a 96-well plate, and an additional three sets of standard samples (each in tetraplicate) were included for quality control and estimation of biological age (GlycanAge, Newcastle upon Tyne, United Kingdom). IgG was isolated from individual plasma samples using a CIM r-Protein G LLD 0.05 mL Monolithic 96-well plate (Sartorius BIA Separation), as described elsewhere (21-23). A volume of 10 μL of the obtained IgG eluates was dried in a vacuum concentrator for the subsequent IgG N-glycan release. IgG N-glycans were released by three-hour incubation with 1.2 U PNGase F enzyme (Promega, Madison, WI, USA) at 37 °C and fluorescently labeled with 8-aminopyrine-1,3,6-trisulfonate (Synchem, Elk Grove Village, IL, USA) in an overnight incubation at 37 °C. Excess reagents were removed by solid-phase extraction on a BioGel P10 (Biorad, Hercules, CA, USA) as a hydrophilic stationary phase (HILIC-SPE). IgG N-glycans were consequently eluted in 450 μL of ultra-pure water and stored at −20 °C until ready for capillary gel electrophoresis with laser-induced fluorescence (CGE-LIF) (23,24). CGE-LIF analysis of IgG N-glycans was performed on ABI3500 Genetic Analyzer (Applied Biosystems, Waltham, MA, USA) equipped with a 50 cm long 8-capillary array (Applied Biosystems), as described elsewhere (23). Briefly, 3 μL of fluorescently labeled IgG N-glycans was mixed with 7 μL of HiDi formamide (Applied Biosystems, USA) and electrokinetically introduced for 12-second into the capillaries filled with POP-7 polymer (Applied Biosystems). IgG N-glycans were separated during the 1000-second run time and a run voltage of 15 kV. The temperature of the capillaries was maintained at 60 °C. Raw data were imported into Empower 3 software (Waters, Milford, MA, USA), where each electropherogram was manually integrated into 27 peaks. The amount of glycans in each peak was expressed as a percentage of the total integrated area. Additionally, biological age was estimated using GlycanAge algorithm, and the corresponding scores (G0, G2, S) were calculated.

### Plasma N-glycome analysis

Plasma samples (10 μL) were first denatured by adding 20 μL of 2% (w/v) sodium dodecyl sulfate (Invitrogen, USA) and heating at 65 °C for 10 minutes. Subsequently, 10 μL of 4% (v/v) Igepal CA-630 (Sigma-Aldrich, Saint Louis, MO, USA) was added, and the mixture was gently agitated for 15 minutes. N-glycans were then released by incubating the samples with 1.2 U of PNGase F (Promega) at 37 °C for 18 hours. The released N-glycans were fluorescently labeled via reductive amination with 2-aminobenzamide (2-AB; Sigma-Aldrich, USA) using 2-picoline borane (Sigma-Aldrich) as the reducing agent. Labeling was performed at 65 °C for 2 hours. For glycan purification, HILIC solid-phase extraction was carried out using a 0.2 μm wwPTFE 96-well filter plate (Pall Corporation, Port Washington, NY, USA) mounted on a vacuum manifold. The filter plate was first preconditioned by sequentially washing with 200 μL of 70% ethanol, 200 μL of ultrapure water, and 200 μL of 96% acetonitrile (ACN). The 2-AB-labeled glycans were then loaded onto the preconditioned plate together with 700 μL of cold 100% ACN, mixed, and incubated for 2 minutes. After three washes with 200 μL of 96% ACN, the purified glycans were eluted with 180 μL of ultrapure water and collected in a clean 0.8 mL round-well plate (Waters). The labeled plasma N-glycan samples were analyzed using a Waters ACQUITY H-Class UHPLC system. Solvent A consisted of 100 mM ammonium formate in water (pH 4.4), while ACN was used as solvent B. A volume of 20 μL of labeled plasma N-glycans was separated on a 150 mm ACQUITY Premier Column (Waters) maintained at 10 °C. Separation was achieved using a linear gradient from 30% to 70% solvent B over 32.50-minute, with a constant flow rate of 0.561 mL/min. Glycans were detected via fluorescence using ACQUITY Premier FLR detectors set to an excitation wavelength of 250 nm and an emission wavelength of 428 nm. Chromatographic peaks were manually integrated using Empower 3 software (Waters), dividing each profile into 39 distinct plasma N-glycan peaks corresponding to known structures, as previously described (24).

### IgA enrichment from plasma and site-specific analysis

IgA was captured from 20 μL plasma using 5 μL of Capture Select IgA-XL Affinity Matrix (Thermo Scientific, Waltham, MA, USA). The affinity matrix was pre-washed with 600 μL of 1 × PBS on Orochem filter plate. Plasma samples were diluted 1:5 with 1 × PBS and added to 30 μL of 1 × PBS left on IgA affinity matrix and incubated on a shaker for an hour. Samples were washed on a vacuum manifold with 600 μL of 1 × PBS and 600 μL of water. IgA was eluted with 100 μL of 100 mM formic acid and centrifuged at 910 × g for 1 minute into a polymerase chain reaction plate. Eluates were dried in a vacuum concentrator at 60 °C for 2 hours, prior to enzymatic digestion. Dried protein was dissolved in 100 μL of 25 mM ammonium bicarbonate, denatured, and reduced with 1 μL of 200 mM DTT by incubation at 60 °C for 30-minute. Iodoacetamide was freshly prepared at 400 mM, and 1.5 μL was added to the samples. Samples were left in the dark for 30-minute. Carbamidomethylation was stopped by adding an excessive amount of 200 mM DTT (3 μL). Trypsin (Sequencing Grade Modified Trypsin, Promega) (0.2 μg) was added to all samples. Incubation was performed for 18 hours at 37 °C to obtain glycopeptides. IgA glycopeptide samples were injected and separated in a 27-minute run on a nanoACQUITY ultra-performance liquid chromatography instrument (Waters) coupled to a compact mass spectrometer (Bruker Daltonics, Billerica, MA, USA). Samples were loaded on a C18 PepMap 100 trap column (300 μm i.d. ×5 mm, particle size 5 μm, Thermo Scientific) and washed with 0.1% trifluoroacetic acid (TFA) at 40 μL/min flow rate for 3 minutes. Separation was performed on a Chromanik SunShell C18 HPLC/UHPLC nano-column (100 μm ×150 mm, particle size 2.6 μm, Uvison Technologies, Sevenoaks, United Kingdom) at 30 °C with a gradient from 100% to 30% of solvent A (0.1% TFA) over 21 minutes, with a flow rate of 1 μL/min. Solvent B was 80% ACN, 0.02% TFA. IgA glycopeptides were baseline separated in clusters according to the peptide backbone. Mass spectra were recorded with two averages at a frequency of 0.5 Hz in a mass range from m/z 500 to m/z 2000. Besides the O-glycosylation site cluster, five N-glycopeptide clusters were detected and quantified: N71(JC) – IIV, N205 (IgA2) – TPL, N340/370tc (IgA1/IgA2) – LAGc (2 elution times), N340/327 (IgA1/IgA2) – LAGy, and N144/131 (IgA1/IgA2) – LSL. Data processing and relative quantification were done with freely available LaCyTools (version 1.1). For the relative quantification, only charge states of glycopeptides that passed quality control parameters: the mass accuracy (between −30 and 30 ppm), deviation from the theoretical isotopic pattern (IPQ, below 30%), and S/N (above 9) of an integrated signal were considered, further resulting in 56 glycopeptides.

## Results

### Overview of measured biochemical parameters

Nineteen biochemical and hormonal blood parameters were measured across three time points: baseline (T0), end-fast (T1) and control (T2). AST levels increased in all participants at T1 and decreased toward baseline or lower values at T2. In contrast, ALT levels also increased at T1 in almost all participants, but showed variable changes at T2, mostly not reverting toward baseline. GGT and ALP demonstrated only minor participant-specific fluctuations across time points. CK and LDH exhibited heterogeneous responses without a consistent group pattern. None of these markers showed significant differences across time points (*P* > 0.05, Friedman test) (Table 1).

**Table 1:** Changes in biochemical parameters across T0, T1, and T2*

	Time period		
Parameter	T0	T1	T2
AST (U/L)	24	33	25
	(16-32)	(22-35)	(16-36)
ALT (U/L)	21	28	25
	(14-31)	(17-33)	(15-44)
GGT (U/L)	35	35^†^	31
	(20-53)	(21-46)	(19-66)
ALP (U/L)	74	65	74
	(59-95)	(60-110)	(57-107)
CK (U/L)	105	201	135
	(47-335)	(52-311)	(51-202)
LDH (U/L)	165	158	159
	(135-190)	(92-175)	(140-210)
Glucose (mmol/L)	5.6	3.8	5.55*
	(4.8-6.7)	(2.3-7.0)	(4.4-6.6)
Total cholesterol (mmol/L)	5.8	6.6	6.3
	(5.3-6.9)	(5.9-8.0)	(5.5-6.9)
Triglycerides (mmol/L)	1.0	1.4	1.1
	(0.9-1.3)	(1.0-1.8)	(0.8-3.0)
HDL-cholesterol (mmol/L)	1.5	1.5	1.5
	(1.1-1.7)	(1.2-1.6)	(1.1-1.5)
LDL-cholesterol (mmol/L)	4.0	4.9	3.9
	(3.3-4.7)	(3.4-5.3)	(3.5-4.5)
Potassium (K^+^) (mmol/L)	4.7	4.5	4.3
	(3.3-5.1)	(4.4-5.0)	(3.8-5.4)
Sodium (Na^+^) (mmol/L)	141	139	141
	(140-143)	(136-143)	(138-142)
C-reactive protein (mg/L)	0.9	2.4	1.3
	(0.2-1.6)	(0.7-6.4)	(0.3-1.5)
TSH (mU/L)	1.55	0.75	1.41
	(1.09-2.35)	(0.45-1.18)	(0.77-1.92)
fT3 (pmol/L)	4.53	3.05	4.59
	(3.96-4.83)	(3.00-3.67)	(3.82-4.71)
fT4 (pmol/L)	13.3	14.5	14.3
	(12.6-14.1)	(11.8-15.7)	(11.5-14.8)
Morning cortisol (nmol/L)	356	417	256
	(267-460)	(215-530)	(198-430)
Insulin (pmol/L)	62.4	34.8	42.5
	(26.1-83.0)	(12.7-100.1)	(15.7-128.0)

*Values are presented as median (min-max).

†Four values instead of five were assessed due to missing information.

Glucose levels decreased at T1 in most participants and reverted toward baseline at T2. Total cholesterol and LDL-cholesterol increased at T1 and decreased toward baseline at T2, while triglycerides showed a similar pattern but with more pronounced individual variability. HDL-cholesterol levels changed variably without a consistent pattern across time points. A statistically significant change was observed for total cholesterol (*P* = 0.021; Friedman test) (Supplemental Figure 1(Supplementary Figure 1)), whereas other glucose and lipid parameters did not significantly change (*P* > 0.05; Friedman test) (Table 2).

**Table 2:** Comparison of biochemical parameter values at T0, T1, and T2

Comparison	Statistic value	*P* value*
AST T0 vs T1 vs T2	5.778	0.056
ALT T0 vs T1 vs T2	5.158	0.076
GGT T0 vs T1 vs T2	0.429	0.807
ALP T0 vs T1 vs T2	2.211	0.331
CK T0 vs T1 vs T2	0.4	0.819
LDH T0 vs T1 vs T2	3.263	0.196
Glucose T0 vs T1 vs T2	2	0.368
Total cholesterol T0 vs T1 vs T2	7.684	0.021
Triglycerides T0 vs T1 vs T2	2.211	0.331
HDL-cholesterol T0 vs T1 vs T2	1.529	0.465
LDL-cholesterol T0 vs T1 vs T2	2.8	0.247
Potassium (K^+^) T0 vs T1 vs T2	0.316	0.854
Sodium (Na^+^) T0 vs T1 vs T2	4.111	0.128
C-reactive protein T0 vs T1 vs T2	7.6	0.022
TSH T0 vs T1 vs T2	10	0.007
fT3 T0 vs T1 vs T2	7.6	0.022
fT4 T0 vs T1 vs T2	0.947	0.623
Morning cortisol T0 vs T1 vs T2	1.6	0.449
Insulin T0 vs T1 vs T2	2.8	0.247

*Friedman test.

Both potassium and sodium demonstrated heterogeneous and mostly minor changes, without a consistent group trend. Electrolytes did not change significantly (*P* > 0.05; Friedman test) (Table 2). CRP levels significantly increased at T1 in all participants and decreased toward baseline at T2 (*P* = 0.022; Friedman test) (Supplemental Figure 2(Supplementary Figure 2), Table 2).

TSH and fT3 demonstrated clear patterns, decreasing at T1 in all participants and increasing toward baseline at T2. fT4 showed minor and inconsistent changes without a particular group trend. Cortisol increased at T1 in most participants and decreased substantially at T2, typically below baseline. Insulin decreased at T1 in most participants and increased at T2, mostly remaining below baseline. Significant changes were observed for TSH (*P* = 0.007; Friedman test) (Supplemental Figure 3(Supplementary Figure 3)) and fT3 (*P* = 0.022; Friedman test) (Supplemental Figure 4(Supplementary Figure 4)), whereas other hormones did not change significantly (*P* > 0.05; Friedman test) (Table 2).

### IgG N-glycans

Galactosylation and bisecting structures exhibited interindividual differences, as well as changes in sialylation across T0, T1, and T2. These changes were reflected in the calculated GlycanAge for each participant.

Participant 3 exhibited the highest GlycanAge (80 years) and a pro-inflammatory profile at T1, characterized by an increase in the agalactosylated (G0) score and decreases in monogalactosylated (G1), digalactosylated (G2), and sialylated (S) scores. In contrast, Participant 2, who also had a GlycanAge of 80 years at baseline, displayed the opposite pattern at T1, with a decrease in G0 and increases in G1, G2, and S scores. Despite these opposing trends at T1, in both participants, all glycan scores returned toward baseline levels at T2, resulting in a stable GlycanAge over time. Participants 4 and 5 showed the same trends in their observed G2, S, and B scores toward a more anti-inflammatory profile, while G0 and G1 exhibited opposite trends between the two participants. These opposing changes are reflected in divergent GlycanAge trajectories, with an approximate 5-year decrease in Participant 5, the most notable change in the data set, and an increase in Participant 4. Participant 1 showed a discrete decrease in GlycanAge, consistent with a lower G0 score compared with other participants, the highest G1 score among all participants, and increased G2 and S scores. The overall primary indices representing pro-inflammation (G0 index) and anti-inflammation (S index and G2 index) are shown in Table 3.

**Table 3:** Primary glycan index median values across T0, T1, and T2*

	Time period		
Index	T0	T1	T2
G0 (pro-inflammatory)	0.277	0.275	0.278
	(0.235-0.465)	(0.234-0.468)	(0.237-0.460)
S (anti-inflammatory)	0.104	0.106	0.105
	(0.076-0.137)	(0.075-0.140)	(0.077-0.140)
G2 (anti-inflammatory)	0.178	0.179	0.180
	(0.105-0.220)	(0.104-0.223)	(0.107-0.222)

*Values are presented as median (min-max).

The G0 index did not significantly change in any of the participants, nor overall (*P* = 0.504; Friedman test). Different values of the G0 index were observed among participants at baseline and remained consistent during the entire process (Supplemental Figure 5(Supplementary Figure 5)).

Similarly, the anti-inflammatory S glycan index (*P* = 0.115; Friedman test) and G2 glycan index (*P* = 0.486; Friedman test) did not considerably change during the study period. Different values of the S and G2 indices were observed among participants at baseline, inverse to the observed G0 levels, and remained consistent during the entire process (Supplemental Figure 6(Supplementary Figure 6) and Supplemental Figure 7(Supplementary Figure 7)).

### IgA glycans

IgA glycosylation showed subtle alterations at three glycosylation sites, reflected in specific glycans. These included fucosylated and sialylated diantennary glycans without the bisection at N205 (IgA2) – TPL, and with additional bisection at N340/327 (IgA1/IgA2) – LAGy. At N144/131 (IgA1/IgA2) – LSL agalactosylated diantennary glycans with bisection were present.

### Plasma glycome

Derived glycan traits – low branching (LB) (*P* = 0.015; Friedman test), highly branching (HB) (*P* = 0.015; Friedman test), trisialylated (S3) (*P* = 0.022; Friedman test), trigalactosylated (G3) (*P* = 0.022; Friedman test), and high-mannose (HM) (*P* = 0.041; Friedman test) changed across the measurement points, indicating pronounced temporal dynamics of the glycomic profile. Between T0 and T1, LB structures increased, HB structures decreased, S3 and G3 decreased, while HM increased. Between T1 and T2, changes were directed into the opposite direction. In addition to changes at the level of derived traits, time-dependent alterations were also observed in the relative abundance of individual glycan structures, including GP15 (A2BG2S1), GP22 (FA2G2S2), GP24 (A3G3S2), GP26 (A3G3S2), GP28 (A3G3S3), GP29 (A3G3S3), GP30 (A3G3S3), GP31 (FA3G3S3), GP35 (FA3F1G3S3), and GP37 (A4G4S4). A complete statistical analysis of all parameters from plasma, IgG, and IgA glycome is available in Supplemental Table 1(Supplementary Table 1).

## Discussion

In this exploratory pilot study, a 72-hour water-only fast in five healthy adults was associated with a biphasic pattern across multiple circulating markers, with changes at the end of the fast (T1) followed by recovery toward baseline at T2. Significant differences were observed in total cholesterol, CRP, TSH, and fT3, and time-dependent changes were also detected across plasma, IgG, and IgA N-glycosylation profiles. Although autophagy was not measured directly, the observed metabolic, hormonal, and glycomic shifts are broadly compatible with the conditions previously described as favorable for autophagy induction during nutrient deprivation. To our knowledge, this is among the first human studies to consider such a broad panel of circulating parameters in the context of fasting and autophagy. Previous fasting studies have evaluated biochemical changes without specifically framing them in the context of autophagy (14,15). Although animal data consistently show fasting-induced autophagy, human studies using direct autophagy markers have produced mixed results, partly because of shorter fasting durations and the difficulty of measuring autophagy in vivo in humans (16,25,26).

**Figure 1:**
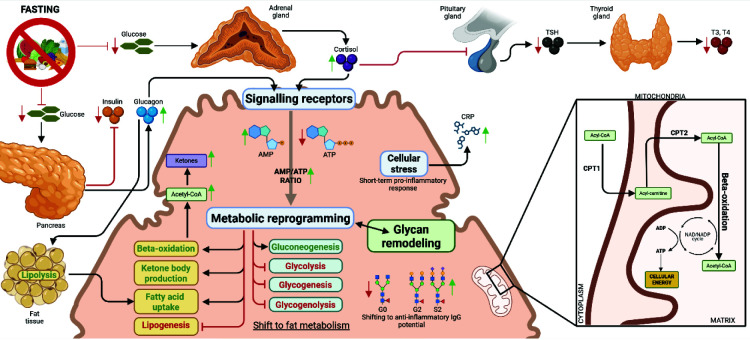
Fasting-induced metabolic changes on the hormonal, cellular, and mitochondrial level (created with Biorender.com).

Autophagy is principally regulated by mTORC1 and AMPK acting through the ULK1 complex, with mTORC1 suppressing and AMPK promoting autophagy in response to nutrient and energy status (27-29). Fasting is therefore expected to favor autophagy through both reduced insulin/mTORC1 signaling and increased AMPK activity (Figure 1).

Consistent with the expected decrease in insulin/mTORC1 signaling, in most participants insulin and glucose concentrations decreased at T1 and increased toward baseline at T2, although neither change reached significance. The direction of these changes is consistent with prior reports during 72-hour fasts (30,31), which mechanistically linked it to reduced mTORC1 activity and increased autophagic flux (20). In our study, most participants showed heterogeneous cortisol responses, with an increase at T1 and a decrease at T2. Both directions are plausible given previously reported time-course dependencies of cortisol during fasting (32,33) and consistent with adaptive mechanisms that prevent sustained hypercortisolism (34). One study described sex-dependent variability, with cortisol substantially decreasing after 48 hours of fasting in healthy women (35). The role of cortisol in modulating autophagy in humans remains complex, tissue-specific, and bidirectional (36,37), and our data are not sufficient to disentangle these effects.

Both TSH and fT3 decreased at T1 and recovered toward baseline at T2, while fT4 changed slightly and inconsistently. This pattern aligns with the well-described “low-T3” state of fasting, in which central and peripheral mechanisms reduce circulating active thyroid hormone to conserve energy (6,15,25,38,39). Of potential relevance to autophagy, T3 has been shown to induce hepatic lipophagy in experimental models (40), which raises the possibility that fluctuations in fT3 during prolonged fasting and refeeding may contribute to the transition between carbohydrate and lipid metabolism; this remains to be tested in human studies with denser sampling.

CRP increased modestly at T1 in all participants and decreased toward baseline at T2, although absolute differences were small and within or near the reference range. Mixed effects of fasting on inflammatory markers have been reported previously, with the overall literature suggesting mild anti-inflammatory effects rather than a pronounced systemic response (41-43). Autophagy and inflammatory signaling are reciprocally linked: autophagy attenuates inflammasome activation by removing damaged mitochondria and other danger signals, and impaired autophagy has been associated with autoimmune and inflammatory disease (44-50). Mild inflammation can, in turn, upregulate autophagy (51). The small CRP increase at T1 is therefore consistent with concurrent activation of autophagy, but our data did not allow us to test this directly.

Lipid changes were characterized by a transient significant rise in total cholesterol at T1 and a similar but non-significant pattern in LDL-cholesterol and triglycerides. HDL-cholesterol remained largely unchanged. A non-significant rise in total cholesterol after short-term fasting has been described previously (14). These changes are compatible with fasting-induced lipolysis, increased hepatic VLDL/LDL production driven by FFA flux, and reduced LDL receptor activity in the setting of low insulin (6,52). Cholesterol also has a recognized role in autophagosome membrane biogenesis through GRAMD1A (53). Therefore, transient elevations in circulating cholesterol may parallel, rather than oppose, an environment favorable to autophagy. Sodium, potassium, and liver and muscle enzymes showed only minor, heterogeneous fluctuations, which were not statistically significant. The small transient increases in AST and ALT at T1 are consistent with previously described benign and reversible hepatic adaptations to prolonged fasting (54,55).

**Figure 2:**
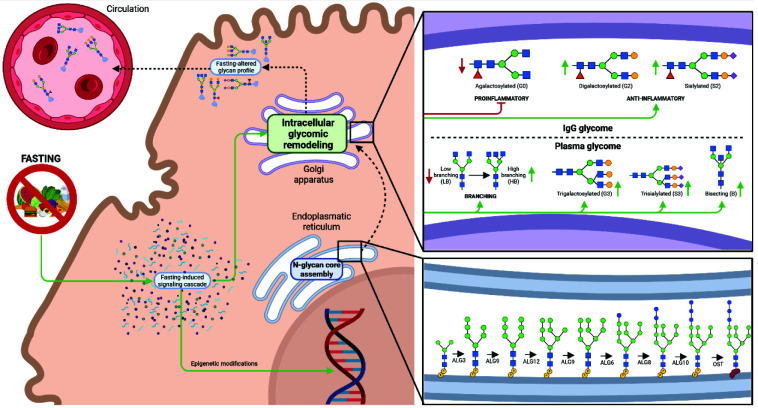
Fasting-induced glycosylation changes on the cellular and circulatory level (created with Biorender.com).

Time-dependent differences were also observed across plasma, IgG, and IgA glycosylation. Although direct evidence linking fasting to glycosylation is lacking, the established metabolic and immune effects of fasting, together with the known role of autophagy in modulating immune and inflammatory responses (56), suggest plausible mechanisms by which glycosylation could be remodeled (Figure 2). In the plasma glycome, significant changes were observed across time points in low- and high-branching, trisialylated, trigalactosylated, and high-mannose traits, as well as several individual peaks (GP15, GP22, GP24, GP26, GP28-GP31, GP35, GP37). Plasma N-glycan patterns are sensitive indicators of inflammatory and metabolic status (57), and changes in plasma glycan traits have been more broadly associated with metabolic and physiological state (58). Two previous dietary-intervention studies reported an increase in low-branching and a decrease in high-branching structures after 8 weeks of a low-calorie diet, and decreased high-branching structures with increased bisecting GlcNAcs after 2 years of caloric restriction (59,60). Our short-term findings are broadly consistent with diet-responsive plasma glycome remodeling, although direct comparisons are limited by the differing durations and intensities of the interventions.

In the IgG glycome, differences in the B-score and in individual peaks suggest modulation of Fc glycosylation, which has been linked to inflammatory status and biological age (61,62). Compared with longer dietary interventions, in which clear shifts toward a less inflammatory IgG glycome have been described (60,63), the changes related to our short fast were subtle and inconsistent across participants. IgA glycosylation showed a distinct pattern that did not mirror the pro-/anti-inflammatory dichotomy seen with IgG, consistent with separate regulatory mechanisms, but the observed differences were subtle and should be regarded as preliminary.

Taken together, the directionally consistent changes in glucose, insulin, fT3/TSH, cortisol, CRP, and lipids at T1; the partial reversal at T2; and the parallel remodeling of plasma and immunoglobulin N-glycomes describe a systemic environment compatible with – though not proof of – increased autophagic activity during a 72-hour fast. The return of most parameters toward baseline at T2 indicates that T1 changes were related to the fasting intervention rather than to random fluctuation, and highlights glycosylation as a dynamic and previously underexplored layer of the systemic fasting response.

Several limitations should be emphasized. The most important is the very small sample size (n = 5, with only one female participant), which limits both statistical power and generalizability. Accordingly, *P* values should be interpreted cautiously and individual trajectories given particular weight. The study is single-arm, without a non-fasting comparison group, although the baseline (T0) and post-refeeding (T2) measurements partly mitigate this. Autophagy was not measured directly; the autophagy-related interpretations require the testing of LC3-II/LC3-I or autophagic flux, along with additional regulators such as leptin and IGF-1, and denser sampling during fasting and refeeding. The discussion of autophagy in this article is therefore framed as hypothesis-generating, based on indirect physiological evidence, and not as a demonstration of autophagy induction in humans.

Our findings may offer a starting point for future research exploring how systemic blood markers could help inform our understanding of autophagy-related processes in humans. Additionally, our fasting protocol appeared to be feasible and well tolerated. Larger studies with more diverse cohorts, direct measurement of autophagy markers, and additional sampling time points are needed to confirm our findings.
