# Supplementary material for: Systemic metabolic, hormonal, and glycomic remodeling during a 72-hour fast in healthy adults: a pilot study
Source: Croat Med J. 2026 Jun;67(3):226–37. doi: 10.3325/cmj.2026.67.226 (PMC13247747; doi:10.3325/cmj.2026.67.226)
Supplement: Supplementary Figure 1 [file CroatMedJ_67_s001.pdf]

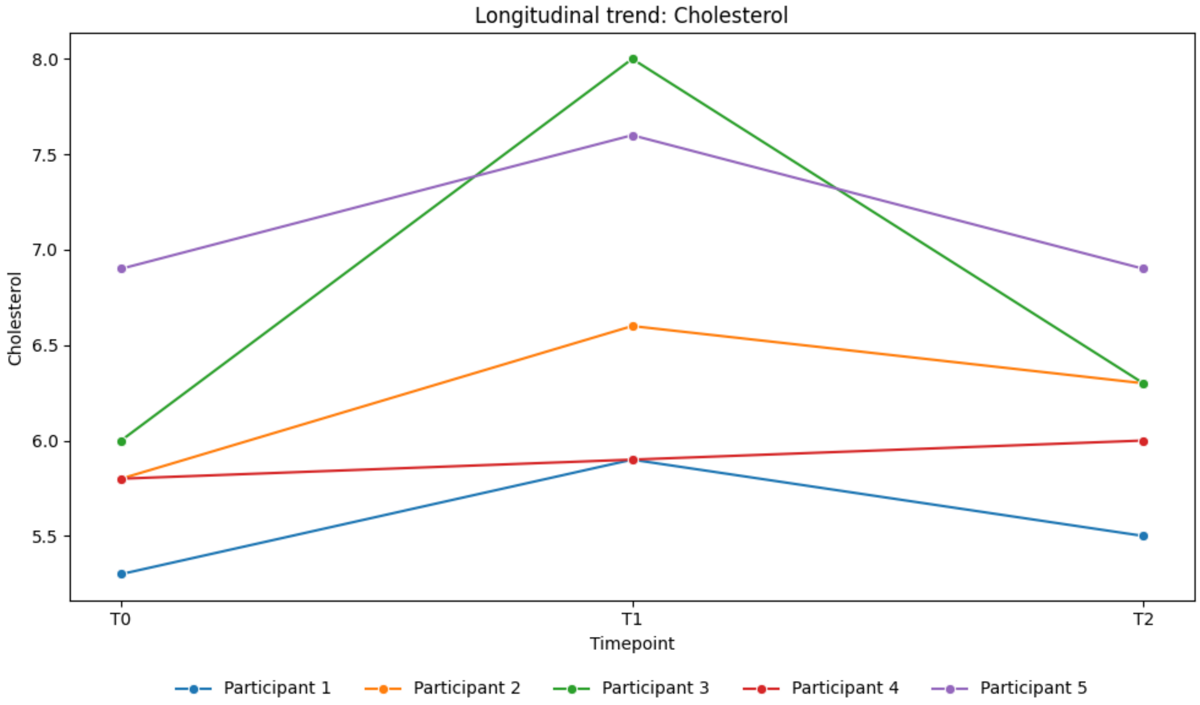

Supplemental Figure 1. Total cholesterol increased at T1 in all participants, with Patient 3 showing the largest rise. At T2, values decreased toward baseline in most participants, with Patient 2 staying above baseline. Patient 4 showed minimal changes across all timepoints.
