# Supplementary material for: Systemic metabolic, hormonal, and glycomic remodeling during a 72-hour fast in healthy adults: a pilot study
Source: Croat Med J. 2026 Jun;67(3):226–37. doi: 10.3325/cmj.2026.67.226 (PMC13247747; doi:10.3325/cmj.2026.67.226)
Supplement: Supplementary Figure 2 [file CroatMedJ_67_s002.pdf]

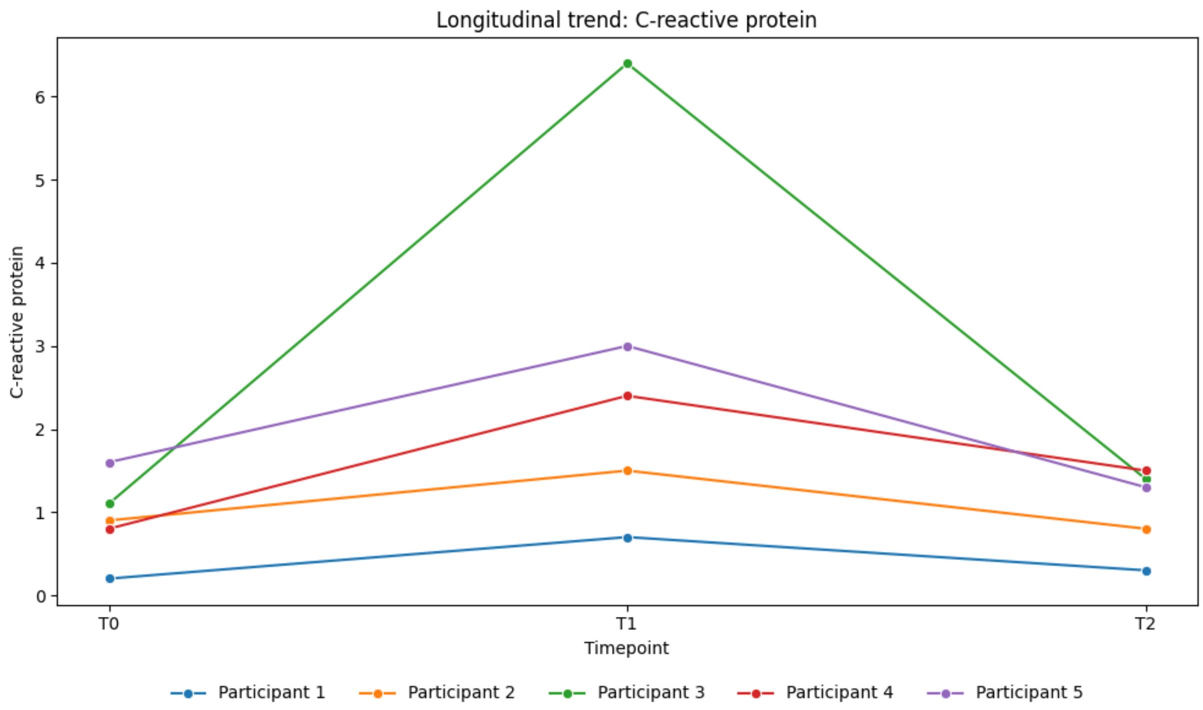

Supplemental Figure 2. CRP levels increased at T1 in all participants and decreased toward baseline at T2. Patient 3 showed the most pronounced increase at T1.
