# Supplementary material for: Systemic metabolic, hormonal, and glycomic remodeling during a 72-hour fast in healthy adults: a pilot study
Source: Croat Med J. 2026 Jun;67(3):226–37. doi: 10.3325/cmj.2026.67.226 (PMC13247747; doi:10.3325/cmj.2026.67.226)
Supplement: Supplementary Figure 3 [file CroatMedJ_67_s003.pdf]

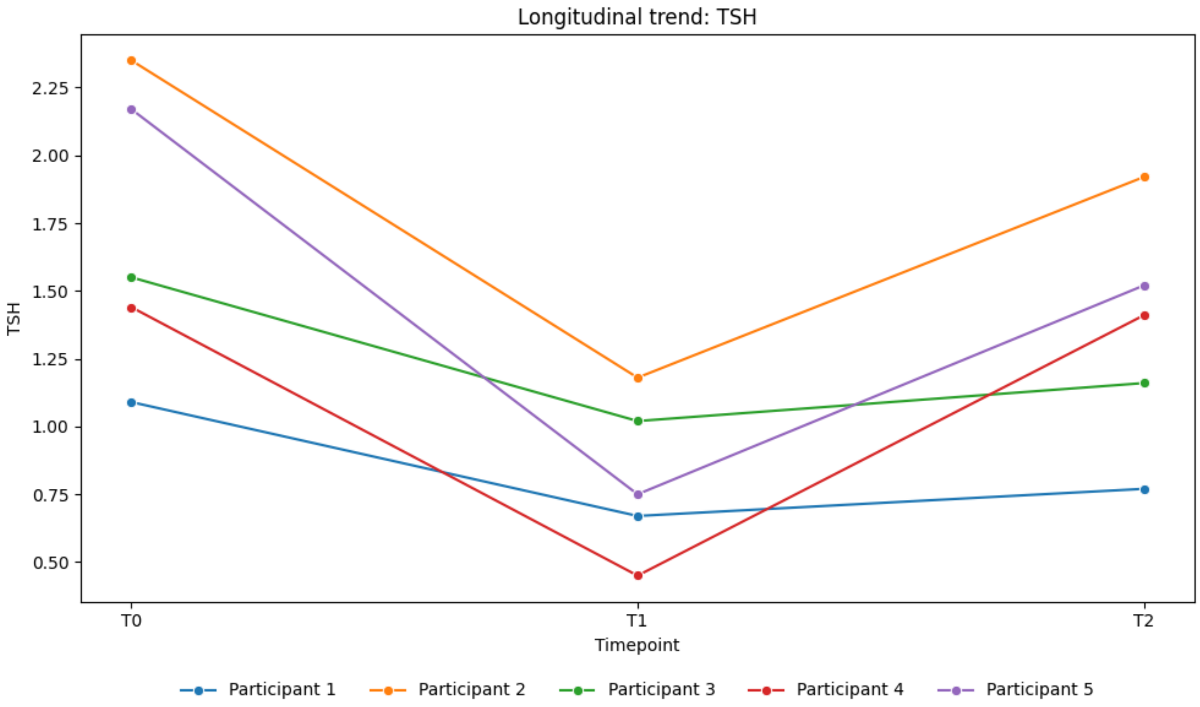

Supplemental Figure 3. TSH levels decreased at T1 in all participants and increased at T2, remaining below baseline in all participants except Patient 4, who nearly returned to baseline.
