# Supplementary material for: Systemic metabolic, hormonal, and glycomic remodeling during a 72-hour fast in healthy adults: a pilot study
Source: Croat Med J. 2026 Jun;67(3):226–37. doi: 10.3325/cmj.2026.67.226 (PMC13247747; doi:10.3325/cmj.2026.67.226)
Supplement: Supplementary Figure 4 [file CroatMedJ_67_s004.pdf]

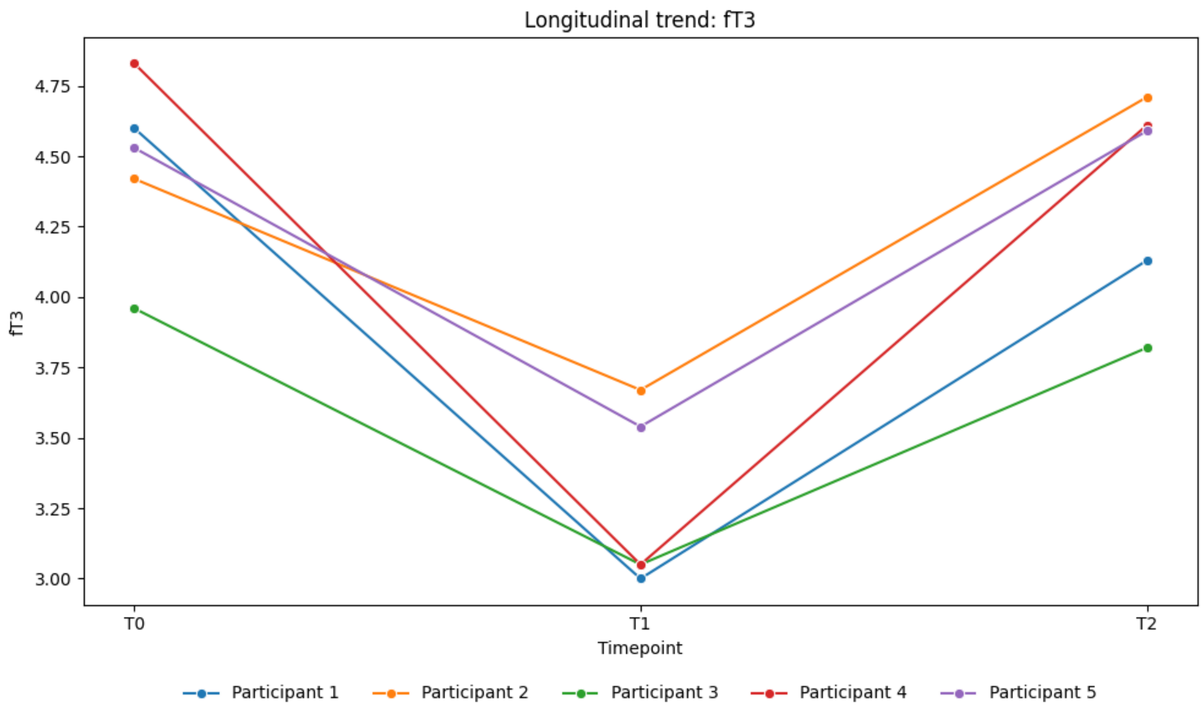

Supplemental Figure 4. fT3 levels decreased at T1 in all participants and increased toward baseline at T2, with Patient 2 exceeding baseline levels at T2.
