# Supplementary material for: Systemic metabolic, hormonal, and glycomic remodeling during a 72-hour fast in healthy adults: a pilot study
Source: Croat Med J. 2026 Jun;67(3):226–37. doi: 10.3325/cmj.2026.67.226 (PMC13247747; doi:10.3325/cmj.2026.67.226)
Supplement: Supplementary Figure 5 [file CroatMedJ_67_s005.pdf]

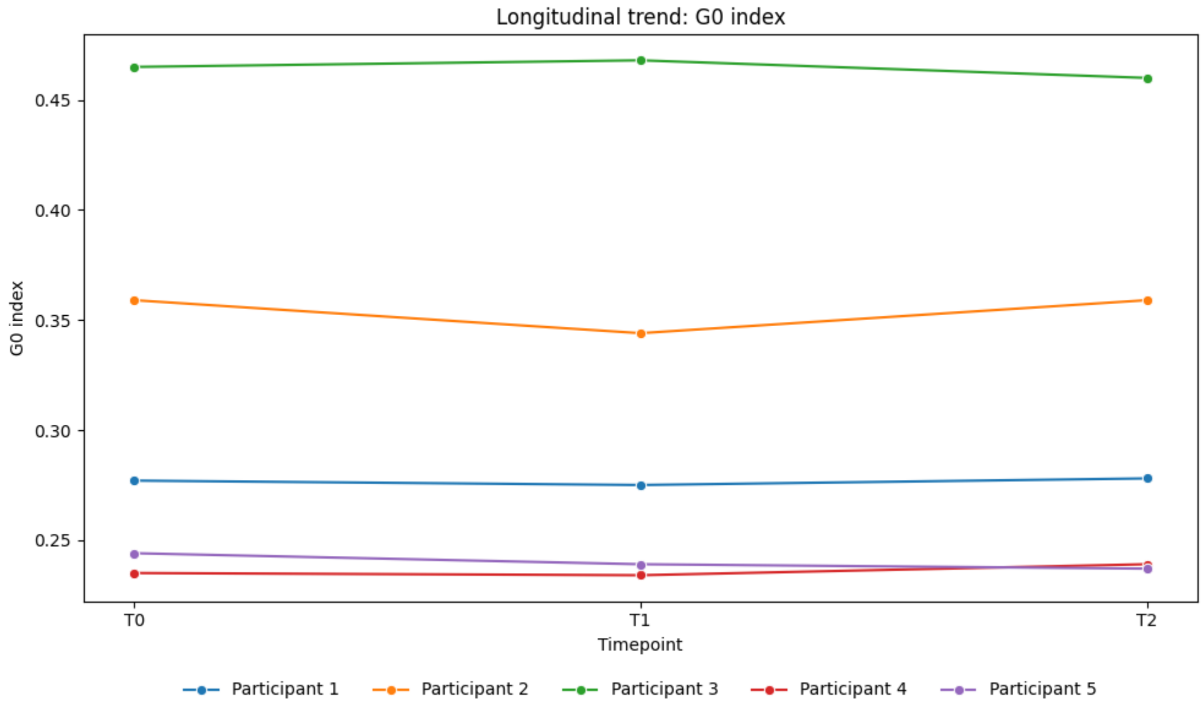

Supplemental Figure 5. The pro-inflammatory G0 glycan index showed no dramatic changes in any of the participants across the T0, T1, and T2. Participant 3 had the highest pro-inflammatory index, followed by participants 2 and 1, while the G0 index values of participants 4 and 5 were low.
