# Supplementary material for: Systemic metabolic, hormonal, and glycomic remodeling during a 72-hour fast in healthy adults: a pilot study
Source: Croat Med J. 2026 Jun;67(3):226–37. doi: 10.3325/cmj.2026.67.226 (PMC13247747; doi:10.3325/cmj.2026.67.226)
Supplement: Supplementary Figure 6 [file CroatMedJ_67_s006.pdf]

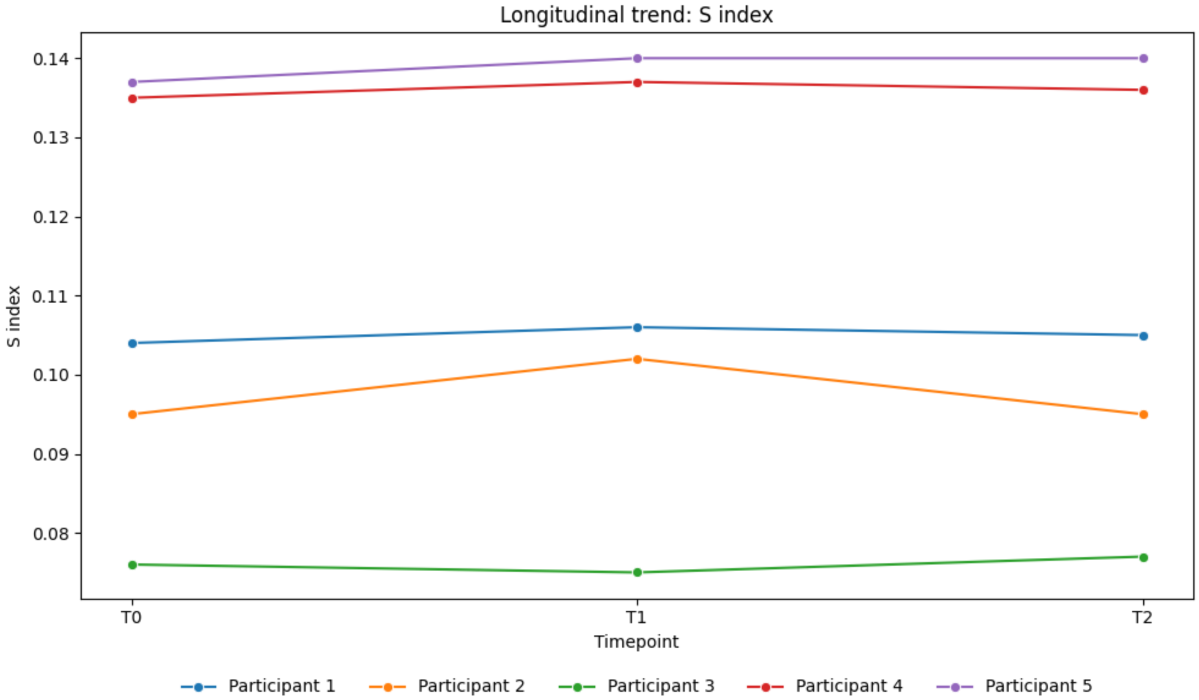

Supplemental Figure 6. The anti-inflammatory S glycan index showed no dramatic changes in any of the participants across the T0, T1, and T2. Participants 4 and 5 had the highest anti-inflammatory index, followed by participants 1 and 2, while the S index value of participant 3 was low.
