# Supplementary material for: Systemic metabolic, hormonal, and glycomic remodeling during a 72-hour fast in healthy adults: a pilot study
Source: Croat Med J. 2026 Jun;67(3):226–37. doi: 10.3325/cmj.2026.67.226 (PMC13247747; doi:10.3325/cmj.2026.67.226)
Supplement: Supplementary Table 1 [file CroatMedJ_67_s008.pdf]

**Supplemental Table 1.** Table of statistical tests performed on plasma, IgG, and IgA glycan parameters

| Comparison | Statistic value (Friedman test) | p-value (* < 0.05) |
|---|---|---|
| **Plasma glycome** | | |
| GP1 T0 vs T1 vs T2 | 1.2 | 0.549 |
| GP2 T0 vs T1 vs T2 | 1.6 | 0.449 |
| GP3 T0 vs T1 vs T2 | 0.4 | 0.819 |
| GP4 T0 vs T1 vs T2 | 1.2 | 0.549 |
| GP5 T0 vs T1 vs T2 | 1.2 | 0.549 |
| GP6 T0 vs T1 vs T2 | 0.4 | 0.819 |
| GP7 T0 vs T1 vs T2 | 4.8 | 0.091 |
| GP8 T0 vs T1 vs T2 | 3.6 | 0.165 |
| GP9 T0 vs T1 vs T2 | 1.6 | 0.449 |
| GP10 T0 vs T1 vs T2 | 1.6 | 0.449 |
| GP11 T0 vs T1 vs T2 | 0 | 1 |
| GP12 T0 vs T1 vs T2 | 5.2 | 0.074 |
| GP13 T0 vs T1 vs T2 | 1.6 | 0.449 |
| GP14 T0 vs T1 vs T2 | 0.4 | 0.819 |
| GP15 T0 vs T1 vs T2 | 6.4 | 0.041 |
| GP16 T0 vs T1 vs T2 | 1.6 | 0.449 |
| GP17 T0 vs T1 vs T2 | 1.2 | 0.549 |
| GP18 T0 vs T1 vs T2 | 5.2 | 0.074 |
| GP19 T0 vs T1 vs T2 | 2.8 | 0.247 |
| GP20 T0 vs T1 vs T2 | 0.4 | 0.819 |
| GP21 T0 vs T1 vs T2 | 2.8 | 0.247 |
| GP22 T0 vs T1 vs T2 | 8.4 | 0.015 |
| GP23 T0 vs T1 vs T2 | 5.2 | 0.074 |
| GP24 T0 vs T1 vs T2 | 8.4 | 0.015 |
| GP25 T0 vs T1 vs T2 | 0 | 1 |
| GP26 T0 vs T1 vs T2 | 8.4 | 0.015 |
| GP27 T0 vs T1 vs T2 | 1.2 | 0.549 |
| GP28 T0 vs T1 vs T2 | 10 | 0.007 |
| GP29 T0 vs T1 vs T2 | 7.6 | 0.022 |
| GP30 T0 vs T1 vs T2 | 8.4 | 0.015 |
| GP31 T0 vs T1 vs T2 | 10 | 0.007 |
| GP32 T0 vs T1 vs T2 | 5.2 | 0.074 |
| GP33 T0 vs T1 vs T2 | 1.6 | 0.449 |
| GP34 T0 vs T1 vs T2 | 5.2 | 0.074 |
| GP35 T0 vs T1 vs T2 | 6.4 | 0.041 |
| GP36 T0 vs T1 vs T2 | 5.2 | 0.074 |
| GP37 T0 vs T1 vs T2 | 7.6 | 0.022 |
| GP38 T0 vs T1 vs T2 | 5.2 | 0.074 |
| GP39 T0 vs T1 vs T2 | 1.6 | 0.449 |
| LB T0 vs T1 vs T2 | 8.4 | 0.015 |
| HB T0 vs T1 vs T2 | 8.4 | 0.015 |
| S0 T0 vs T1 vs T2 | 1.2 | 0.549 |
| S1 T0 vs T1 vs T2 | 2.8 | 0.247 |

| | | |
|---|---|---|
| S2 T0 vs T1 vs T2 | 0.4 | 0.819 |
| S3 T0 vs T1 vs T2 | 7.6 | 0.022 |
| S4 T0 vs T1 vs T2 | 2.8 | 0.247 |
| G0 T0 vs T1 vs T2 | 1.2 | 0.549 |
| G1 T0 vs T1 vs T2 | 1.2 | 0.549 |
| G2 T0 vs T1 vs T2 | 2.8 | 0.247 |
| G3 T0 vs T1 vs T2 | 7.6 | 0.022 |
| G4 T0 vs T1 vs T2 | 5.2 | 0.074 |
| HM T0 vs T1 vs T2 | 6.4 | 0.041 |
| B T0 vs T1 vs T2 | 1.6 | 0.449 |
| CF T0 vs T1 vs T2 | 1.6 | 0.449 |
| AF T0 vs T1 vs T2 | 1.2 | 0.549 |
| **IgG glycome** | | |
| P1 T0 vs T1 vs T2 | 0.4 | 0.819 |
| P2 T0 vs T1 vs T2 | 2.8 | 0.247 |
| P3 T0 vs T1 vs T2 | 1.6 | 0.449 |
| P4 T0 vs T1 vs T2 | 4.8 | 0.091 |
| P5 T0 vs T1 vs T2 | 0.4 | 0.819 |
| P6 T0 vs T1 vs T2 | 5.2 | 0.074 |
| P7 T0 vs T1 vs T2 | 1.6 | 0.449 |
| P8 T0 vs T1 vs T2 | 5.2 | 0.074 |
| P9 T0 vs T1 vs T2 | 0.4 | 0.819 |
| P10 T0 vs T1 vs T2 | 5.2 | 0.074 |
| P11 T0 vs T1 vs T2 | 1.6 | 0.449 |
| P12 T0 vs T1 vs T2 | 7.6 | 0.022 |
| P13 T0 vs T1 vs T2 | 2.8 | 0.247 |
| P14 T0 vs T1 vs T2 | 0.4 | 0.819 |
| P15 T0 vs T1 vs T2 | 1.2 | 0.549 |
| P16 T0 vs T1 vs T2 | 1.2 | 0.549 |
| P17 T0 vs T1 vs T2 | 0.4 | 0.819 |
| P18 T0 vs T1 vs T2 | 2.8 | 0.247 |
| P19 T0 vs T1 vs T2 | 1.2 | 0.549 |
| P20 T0 vs T1 vs T2 | 5.2 | 0.074 |
| P21 T0 vs T1 vs T2 | 3.6 | 0.165 |
| P22 T0 vs T1 vs T2 | 1.6 | 0.449 |
| P23 T0 vs T1 vs T2 | 8.4 | 0.015 |
| P24 T0 vs T1 vs T2 | 0.4 | 0.819 |
| P25 T0 vs T1 vs T2 | 7.6 | 0.022 |
| P26 T0 vs T1 vs T2 | 1.2 | 0.549 |
| P27 T0 vs T1 vs T2 | 2.8 | 0.247 |
| G0yourscore T0 vs T1 vs T2 | 1.368 | 0.504 |
| G1yourscore T0 vs T1 vs T2 | 0 | 1 |
| G2yourscore T0 vs T1 vs T2 | 1.444 | 0.486 |
| Syourscore T0 vs T1 vs T2 | 4.333 | 0.115 |
| Byourscore T0 vs T1 vs T2 | 8.375 | 0.015 |
| glycanage T0 vs T1 vs T2 | 2.364 | 0.307 |
| **IgA glycome** | | |

| | | |
|---|---|---|
| TPL_N4H4F1 T0 vs T1 vs T2 | 1.6 | 0.449 |
| TPL_N4H5S1F1 T0 vs T1 vs T2 | 4.8 | 0.091 |
| TPL_N4H5S2F1 T0 vs T1 vs T2 | 6.4 | 0.041 |
| TPL_N5H5F1 T0 vs T1 vs T2 | 0.4 | 0.819 |
| TPL_N5H5S1F1 T0 vs T1 vs T2 | 1.2 | 0.549 |
| TPL_N5H5S2F1 T0 vs T1 vs T2 | 2.8 | 0.247 |
| IIV_N3H5S1F1 T0 vs T1 vs T2 | 1.2 | 0.549 |
| IIV_N4H5S1F1 T0 vs T1 vs T2 | 0 | 1 |
| IIV_N4H5S2 T0 vs T1 vs T2 | 0.4 | 0.819 |
| IIV_N4H5S2F1 T0 vs T1 vs T2 | 0.4 | 0.819 |
| LAGca_N4H5S1F1 T0 vs T1 vs T2 | 1.2 | 0.549 |
| LAGca_N4H5S2F1 T0 vs T1 vs T2 | 0.4 | 0.819 |
| LAGca_N5H5S1F1 T0 vs T1 vs T2 | 1.2 | 0.549 |
| LAGca_N5H5S2F1 T0 vs T1 vs T2 | 0.4 | 0.819 |
| LAGcb_N3H4 T0 vs T1 vs T2 | 0.4 | 0.819 |
| LAGcb_N4H4S1F1 T0 vs T1 vs T2 | 1.2 | 0.549 |
| LAGcb_N4H5S1F1 T0 vs T1 vs T2 | 0.4 | 0.819 |
| LAGcb_N4H5S2F1 T0 vs T1 vs T2 | 0.4 | 0.819 |
| LAGcb_N5H5S1F1 T0 vs T1 vs T2 | 1.2 | 0.549 |
| LAGcb_N5H5S2F1 T0 vs T1 vs T2 | 0.4 | 0.819 |
| LAGcb_N6H6S3F1 T0 vs T1 vs T2 | 1.2 | 0.549 |
| LAGy_N4H5S1 T0 vs T1 vs T2 | 0.4 | 0.819 |
| LAGy_N4H5S1F1 T0 vs T1 vs T2 | 0.4 | 0.819 |
| LAGy_N4H5S2 T0 vs T1 vs T2 | 2.8 | 0.247 |
| LAGy_N4H5S2F1 T0 vs T1 vs T2 | 2.8 | 0.247 |
| LAGy_N5H4S1F1 T0 vs T1 vs T2 | 0.4 | 0.819 |
| LAGy_N5H5S1F1 T0 vs T1 vs T2 | 6.4 | 0.041 |
| LAGy_N5H5S2F1 T0 vs T1 vs T2 | 7.6 | 0.022 |

| | | |
|---|---|---|
| LAGy_N5H6S2F1 T0 vs T1 vs T2 | 2.8 | 0.247 |
| LSL_N3H5S1F1 T0 vs T1 vs T2 | 1.2 | 0.549 |
| LSL_N4H4S1 T0 vs T1 vs T2 | 2.8 | 0.247 |
| LSL_N4H5 T0 vs T1 vs T2 | 0.4 | 0.819 |
| LSL_N4H5S1 T0 vs T1 vs T2 | 1.2 | 0.549 |
| LSL_N4H5S2 T0 vs T1 vs T2 | 3.6 | 0.165 |
| LSL_N5H3 T0 vs T1 vs T2 | 8.4 | 0.015 |
| LSL_N5H4 T0 vs T1 vs T2 | 1.6 | 0.449 |
| LSL_N5H4S1 T0 vs T1 vs T2 | 0.4 | 0.819 |
| LSL_N5H5 T0 vs T1 vs T2 | 0 | 1 |
| LSL_N5H5S1 T0 vs T1 vs T2 | 1.6 | 0.449 |
| LSL_N5H5S2 T0 vs T1 vs T2 | 0.4 | 0.819 |
| HYT_N3H3S3 T0 vs T1 vs T2 | 1.2 | 0.549 |
| HYT_N4H3S2 T0 vs T1 vs T2 | 2.8 | 0.247 |
| HYT_N4H3S3 T0 vs T1 vs T2 | 3.6 | 0.165 |
| HYT_N4H4 T0 vs T1 vs T2 | 1.2 | 0.549 |
| HYT_N4H4S1 T0 vs T1 vs T2 | 1.6 | 0.449 |
| HYT_N4H4S2 T0 vs T1 vs T2 | 2.8 | 0.247 |
| HYT_N4H4S3 T0 vs T1 vs T2 | 1.2 | 0.549 |
| HYT_N4H4S4 T0 vs T1 vs T2 | 0.4 | 0.819 |
| HYT_N5H3S2 T0 vs T1 vs T2 | 2.8 | 0.247 |
| HYT_N5H3S3 T0 vs T1 vs T2 | 2.8 | 0.247 |
| HYT_N5H4S1 T0 vs T1 vs T2 | 0 | 1 |
| HYT_N5H4S2 T0 vs T1 vs T2 | 3.6 | 0.165 |
| HYT_N5H4S3 T0 vs T1 vs T2 | 1.2 | 0.549 |
| HYT_N5H5S1 T0 vs T1 vs T2 | 0.4 | 0.819 |
| HYT_N5H5S2 T0 vs T1 vs T2 | 2.8 | 0.247 |
| HYT_N5H5S3 T0 vs T1 vs T2 | 1.6 | 0.449 |
| TPL__Stot T0 vs T1 vs T2 | 1.2 | 0.549 |
| TPL__Alow T0 vs T1 vs T2 | 2.8 | 0.247 |
| TPL__Ahigh T0 vs T1 vs T2 | 2.8 | 0.247 |

| | | |
|---|---|---|
| TPL__Slow T0 vs T1 vs T2 | 1.2 | 0.549 |
| IIV__Ftot T0 vs T1 vs T2 | 0.4 | 0.819 |
| IIV__Alow T0 vs T1 vs T2 | 1.2 | 0.549 |
| LAGca__Alow T0 vs T1 vs T2 | 1.2 | 0.549 |
| LAGca__Ahigh T0 vs T1 vs T2 | 1.2 | 0.549 |
| LAGcb__Ftot T0 vs T1 vs T2 | 0.4 | 0.819 |
| LAGcb__Stot T0 vs T1 vs T2 | 0.4 | 0.819 |
| LAGcb__Alow T0 vs T1 vs T2 | 0.4 | 0.819 |
| LAGcb__Ahigh T0 vs T1 vs T2 | 2.8 | 0.247 |
| LAGcb__Slow T0 vs T1 vs T2 | 1.2 | 0.549 |
| LAGcb__Shigh T0 vs T1 vs T2 | 1.2 | 0.549 |
| LAGy__Ftot T0 vs T1 vs T2 | 2.8 | 0.247 |
| LAGy__Alow T0 vs T1 vs T2 | 5.2 | 0.074 |
| LAGy__Ahigh T0 vs T1 vs T2 | 5.2 | 0.074 |
| LSL__Ftot T0 vs T1 vs T2 | 1.2 | 0.549 |
| LSL__Stot T0 vs T1 vs T2 | 2.8 | 0.247 |
| LSL__Alow T0 vs T1 vs T2 | 1.2 | 0.549 |
| LSL__Ahigh T0 vs T1 vs T2 | 1.2 | 0.549 |
| LSL__Slow T0 vs T1 vs T2 | 2.8 | 0.247 |
| HYT__N3 T0 vs T1 vs T2 | 1.2 | 0.549 |
| HYT__N4 T0 vs T1 vs T2 | 1.2 | 0.549 |
| HYT__N5 T0 vs T1 vs T2 | 1.2 | 0.549 |
| HYT__H3 T0 vs T1 vs T2 | 2.8 | 0.247 |
| HYT__H4 T0 vs T1 vs T2 | 1.2 | 0.549 |
| HYT__H5 T0 vs T1 vs T2 | 1.6 | 0.449 |
| HYT__S1 T0 vs T1 vs T2 | 1.6 | 0.449 |
| HYT__S2 T0 vs T1 vs T2 | 1.2 | 0.549 |
| HYT__S3 T0 vs T1 vs T2 | 0.4 | 0.819 |
| HYT__S4 T0 vs T1 vs T2 | 0.4 | 0.819 |
| HYT__Hdef T0 vs T1 vs T2 | 0.4 | 0.819 |
